# In Pursuit of Adult Progenitors of Thymic Epithelial Cells

**DOI:** 10.3389/fimmu.2021.621824

**Published:** 2021-02-25

**Authors:** Tatsuya Ishikawa, Nobuko Akiyama, Taishin Akiyama

**Affiliations:** ^1^Laboratory of Immune Homeostasis, RIKEN Center for Integrative Medical Sciences, Yokohama, Japan; ^2^Graduate School of Medical Life Science, Yokohama City University, Yokohama, Japan; ^3^Laboratory for Immunogenetics, RIKEN Center of Integrative Medical Sciences, Yokohama, Japan

**Keywords:** thymus, thymic epithelial cells, immunodeficiency, immune dysregulation, single cell RNA-sequencing

## Abstract

Peripheral T cells capable of discriminating between self and non-self antigens are major components of a robust adaptive immune system. The development of self-tolerant T cells is orchestrated by thymic epithelial cells (TECs), which are localized in the thymic cortex (cortical TECs, cTECs) and medulla (medullary TECs, mTECs). cTECs and mTECs are essential for differentiation, proliferation, and positive and negative selection of thymocytes. Recent advances in single-cell RNA-sequencing technology have revealed a previously unknown degree of TEC heterogeneity, but we still lack a clear picture of the identity of TEC progenitors in the adult thymus. In this review, we describe both earlier and recent findings that shed light on features of these elusive adult progenitors in the context of tissue homeostasis, as well as recovery from stress-induced thymic atrophy.

## Introduction

Once regarded as a vestigial organ that had lost its function during evolution, the thymus is now recognized as a primary lymphoid organ that performs irreplaceable functions in differentiation and selection of self-tolerant T cells (1). The thymus “educates” T cells by allowing those that possess a T-cell-receptor capable of interacting with major histocompatibility complex class I or II (MHCI and MHCII) molecules to survive, while eliminating those that recognize self-antigens presented by MHCI or MHCII molecules. This event is orchestrated by two types of thymic epithelial cells (TECs) that reside in the thymic cortex (cortical thymic epithelial cells, cTECs) and medulla (medullary thymic epithelial cells, mTECs). Functionally, cTECs are mainly required for lineage commitment, expansion, and positive selection of thymocytes, while mTECs promote negative selection of self-reactive T cells or promote their diversification into the regulatory T cell lineage by ectopically expressing self-derived tissue-specific antigens (2–4). The expression of tissue-specific antigens is partially regulated by the autoimmune regulator (AIRE), the mutation and dysfunction of which contribute to severe autoimmune diseases (5).

In the past several years, intense effort has focused on understanding the development of TECs, and bipotent and unipotent progenitors of cTECs and mTECs have been rigorously studied. The presence of embryonic bipotent progenitors of cTECs and mTECs was evidenced by transplanting a single early embryonic TEC (day 12.5) into the fetal thymus to generate both cTECs and mTECs (6), and by neonatal reactivation of developmentally arrested fetal bipotent progenitors (7). These embryonic bipotent progenitors can be identified by expression of placenta-expressed transcript 1 (PLET1), and resemble the phenotype of cTECs expressing CD205, β5t, and IL-7 (8–12). Although evidence regarding embryonic unipotent progenitors of cTECs is limited (13), equivalent progenitors of mTECs have been well-studied (14–16). Overall, it is most likely that mTEC progenitors and cTECs are derived from bipotent progenitors in the embryonic thymus, although mechanisms underlying fate decisions of bipotent TEC progenitors remain elusive.

In contrast to embryonic TEC progenitors, little is known about the corresponding progenitors that maintain thymic tissue function in adults. In this review, we first highlight some reported properties of adult progenitors under homeostatic conditions, and then review how the putative adult progenitors contribute to thymic regeneration, from both the cellular and molecular perspectives, following stress-induced damage to the thymus.

## Adult TEC Progenitors Under Homeostatic Conditions

*In vivo* cell labeling and ablation studies suggested that TECs are able to undergo turnover in the adult thymus (14, 17, 18). Adult progenitors or stem cells of TECs should exist to maintain the steady-state functions of the thymus so as to repopulate the periphery with immunologically competent T cells. In this section, we describe some recent findings on the identity of these elusive adult TEC progenitors under steady-state conditions.

### Differences Between Embryonic and Adult TEC Progenitors

Earlier studies have suggested that some molecular features of TEC progenitors may differ between embryonic and adult thymus. Although mTEC-restricted progenitors are enriched in cells expressing the embryonic stem cell marker, SSEA-1, within the CLAUDIN-3/4-positive (CLD3/4^hi^) population of the fetal thymus (16), these fractions appear to lose their self-replicating capacity in the adult thymus (16). Most SSEA-1^+^ CLD3/4^hi^ TECs in the adult thymus are MHCII^lo/−^ cells and express keratin 10, a marker of terminally differentiated mTECs known as Post-AIRE mTECs (16, 19–22). Moreover, unlike β5t^+^ embryonic bipotent progenitors, adult β5t^+^ cells contribute minimally to maintenance of TECs (23, 24). In light of these findings, Ohigashi et al. (23) argued that although adult progenitors are derived from embryonic β5t^+^ bipotent progenitors, they develop into mTEC-restricted SSEA-1^+^ CLD3/4^hi^ progenitors after losing β5t expression.

### Adult Bipotent Progenitors

The existence of an adult bipotent progenitor capable of supplying cTECs and mTECs was tested using the thymic reaggregation/transplantation approach (25, 26). Ulyanchenko et al. demonstrated that TECs expressing LY51 (a marker of cTECs) and PLET1 had progenitor activities. Notably, the activity of bipotent progenitors generating both cTECs and mTECs were present in the MHCII^+^ fraction of Ly51^+^PLET1^+^ TECs (hereafter referred to as PLET1^+^ TECs) (25). On the other hand, Wong et al. (26) proposed that bipotent progenitors are present in a subset of TECs expressing low levels of MHCII and LY51 and lacking the mTEC marker UEA-1 ligand (referred to as TEC^lo^). They both found that bipotent progenitors were present in UEA-1-negative TEC fractions, and that they express surface LY51 and *Pax1* mRNA, suggesting their similarity to cTECs. Unfortunately, there are some discrepancies between these studies. For example, PLET1^+^ TECs are enriched in the MHCII^hi^ fraction, but TEC^lo^ belongs to MHCII^lo^ fraction (25, 26). PLET1^+^ TECs comprise <1% of all TECs, and limiting dilution analysis suggested their bipotency at nearly clonal resolution (25). In contrast, TEC^lo^ comprises about 20% of all TECs (26). Therefore, it may be possible that both unipotent cTEC and mTEC progenitors could be present in TEC^lo^ (25, 26). Importantly, as both studies verified their differentiation potential using reaggregation with fetal thymic cells, such conditions may not be suitable to address adult progenitors. Moreover, details of experimental conditions for thymic reaggregation differed slightly between two studies, which may explain the discrepancy. *In vivo* fate mapping needs to be performed in the adult thymus to evaluate their physiological fate.

### A Subset of mTEC^lo^ Cells Represents mTEC Lineage-Restricted Adult Progenitors

Previously, mTECs were categorized as mTEC^lo^ or mTEC^hi^, depending on expression levels of AIRE, CD80, and MHCII. mTEC^lo^ cells expressing lower levels of AIRE, CD80, and MHCII have been considered as an immature stage of mTEC^hi^ (17, 27–29). However, recent findings demonstrated that the mTEC^lo^ fraction contains multiple subsets. Several studies showed that AIRE^+^ mTEC^hi^ can further differentiate into mTECs with lower expression of AIRE, CD80, and MHCII (Post-AIRE mTECs), which are included in the mTEC^lo^ fraction (19–22). Moreover, Lucas et al. (30) demonstrated that the mTEC^lo^ fraction can be segregated by expression of the chemokine, CCL21, into CD104^+^ CCL21^+^ and CD104^−^ CCL21^−^ subsets. Since CCL21 recruits positively selected thymocytes to the thymic medulla, the CD104^+^ CCL21^+^ mTEC^lo^ subset may be considered functionally mature cells (31). Onder et al. (32) reported that within CD80^−^ TECs, there is a population of podoplanin (Pdpn)-expressing mTEC-restricted progenitors localized in the cortical-medullary junction (junctional TECs). In summary, it is likely that a limited population of mTEC^lo^ cells should be unipotent progenitors of mTEC^hi^.

### New Insights Gained From Single-Cell RNA-Sequencing Studies

Recent progress in single-cell RNA-sequencing (scRNA-seq) technology has uncovered a previously unknown degree of heterogeneity among TECs and has provided new insights into both the developmental pathway and mechanism of TECs, especially mTECs under homeostatic conditions (33–36). Based on the cell type clusters obtained from scRNA-seq results, Bornstein *et al*. categorized mTECs into four subsets: mTEC I, mTEC II, mTEC III, and a newly identified mTEC IV or tuft cells with chemosensory properties (33, 35). With respect to previous mTEC classifications, the mTEC I, mTEC II, and mTEC III subsets are equivalent to CCL21^+^ mTEC^lo^, AIRE^+^ mTEC^hi^, and Post-AIRE mTEC subsets, respectively. Notably, Lucas et al. (30) showed that DCLK1^+^ mTEC IV/tuft cells are enriched in the CD104^−^ CCL21^−^ mTEC^lo^ subset. Additionally, Dhalla et al. (34) used scRNA-seq to more deeply interrogate mTEC heterogeneity. These authors identified a “Proliferating mTEC” cluster that seemed to bridge the clusters representing mature AIRE^+^ mTEC^hi^ and CCL21^+^ mTEC^lo^ (34). Cells in the “Proliferating mTEC” cluster exhibited upregulation of genes involved in proliferation, such as *Mki67*, and expressed *Aire*, suggesting that it could represent proliferating mTECs previously reported within mTEC^hi^ (14, 26, 34). The trajectory of diffusion pseudotime analysis suggested that cells in the “Proliferating mTEC” cluster could act as bipotent mTEC progenitors that differentiate into both AIRE^+^ mTEC^hi^ and CCL21^+^ mTEC^lo^ lineages (34). However, analysis of the same data using RNA velocity, a different trajectory method that relies on pre- and post-spliced RNA reads (37), produced conflicting results. The latter analysis indicated that rather than differentiating into the CCL21^+^ mTEC^lo^ cluster, the “Proliferating mTEC” cluster seemed to be derived from CCL21^+^ mTEC^lo^ and junctional TEC clusters (34). In a different study, Baran-Gale et al. (38) conducted scRNA-seq of mouse TECs throughout the 1st year of life and studied their various trajectories using genetic fate mapping under control of the β5t promoter. They also identified a cluster equivalent to the “Proliferating mTECs” (38). However, their diffusion pseudotime analysis failed to suggest that the “Proliferating mTEC” cluster was positioned at the branch point between mTEC differentiation into AIRE^+^ mTEC^hi^ and CCL21^+^ mTEC^lo^ lineages (38). Instead, they showed that the “Intertypical TEC” cluster, which encompassed cTEC, CCL21^+^ mTEC^lo^, and junctional TEC, could bifurcate into two mTEC trajectories that both progressed toward AIRE^+^ mTEC^hi^ via the “Proliferating mTEC” cluster (38). In a more recent study, Wells et al. (39) showed that the “TAC-TEC” cluster, a cluster equivalent to the “Proliferating mTECs,” could give rise to both AIRE^+^ mTEC^hi^ and CCL21^+^ mTEC^lo^ lineages, using RNA velocity. In summary, *in silico* predictions of mTEC differentiation dynamics deduced from scRNA-seq data were split. These discrepancies could be due to differences in cell coverages and sequencing depths detected using different scRNA-seq methods (Table 1). For instance, low numbers of detected mRNA species could affect RNA velocity analysis, which relies on detection of unspliced mRNAs occupying 15–25% of total sequencing reads in scRNA data (37).

**Table 1:** Comparison of scRNA-seq methods (FACS sorted cells in wells or droplet-based), cell numbers used after quality control, sequencing depths, and methods of trajectory analysis used by different scRNA-seq studies from postnatal thymus glands of wild-type mice under homeostasis.

	**scRNA-seq method**	**Number of cells used in analysis**	**Sequencing depth per cell**	**Trajectory analysis**
Bornstein et al. (33)	MARS-seq (FACS)	1,825 CD45^−^ cells and 1,716 TECs	Median of 1,711 UMIs	None
Dhalla et al. (34)	10X Genomics (Droplet)	6,894 mTECs	Median of 1,830 genes	Diffusion pseudotime RNA velocity
Baren-Gale et al. (38)	SMART-Seq2 (FACS)	2,327 TECs	Not mentioned	Diffusion pseudotime
Wells et al. (39)	10X Genomics (Droplet)	2,434 TECs	200–7,500 genes	RNA velocity

Notably, the “Intertypical TEC” cluster contained PLET1^+^ TECs and expressed markers associated with the bipotent TEC^lo^ progenitors, which were identified by cytometry-based analysis as described above (25, 26, 38). Diffusion pseudotime analysis showed that the “Intertypical TEC” cluster could not only contribute to the mTEC lineage, but also to the cTEC lineage, suggesting its bipotency (38). Additionally, these authors suggested that the aging “Intertypical TEC” cluster displayed features of progressive quiescence, and that it could arise from either β5t^+^ or β5t^−^ progenitors independently (38). This proposal contradicts the argument put forth by Ohigashi et al. (23) that β5t^+^ and β5t^−^ progenitors possess a precursor-product relationship.

Importantly, all of the clusters described above, which are identified in mouse analyses, can also be identified in scRNA-seq data obtained from the human thymus, indicating that the cluster-based classification is not restricted to mice (38, 40). Nevertheless, we await experimental verification of the existence and function of putative adult progenitors inferred from computationally defined clusters.

## Adult TEC Progenitors During Recovery From Stress-Induced Damage

Adult progenitors would be integral not only to maintaining tissue homeostasis, but also to recovery of the thymus from stress-induced damage. In the following sections, we describe how putative adult TEC progenitors could contribute to thymic recovery at both the cellular and molecular levels, based on studies using mouse models that mimic insults.

### Repair Potential of the Damage-Sensitive Thymus

The thymus is extremely sensitive to damage and exposure to acute or chronic insults results in a pronounced decline in cellularity, a phenomenon known as thymic atrophy (41, 42). For example, we recently demonstrated thymic atrophy displayed by mice under microgravity (0 g) conditions during spaceflight, which was partially mitigated by exposure to 1 g during spaceflight (43). After resolution of acute insults such as infections, cytoreductive therapies, and emotional and physical discomfort, the thymus is able to regenerate, although its capacity declines with age (41, 42). To study this endogenous thymic regeneration, researchers have employed viral and bacterial infections, sub-lethal irradiation, and synthetic corticosteroid injections to model acute insults (44–50). Chronic insults, such as aging, hamper the ability of the thymus to regenerate, but age-induced defects in recovery can be transiently reversed by ablation of sex steroids (51–53). This can be explained by the ability of sex hormones to induce apoptosis and to inhibit proliferation of developing T cells (54, 55). In fact, the regenerative capacity of the thymus was known well before its function as a lymphoid organ was discovered (56, 57).

### Cellular Mechanisms of TEC Regeneration

Several studies have shed light on cellular mechanisms of TEC regeneration, which appear to initiate from the putative adult progenitors. Using an irradiation-induced stress model, we recently performed a quantitative analysis of TEC regeneration and its mathematical modeling (58). We showed that full recovery was reached earlier by cTECs than mTECs, and that mTEC recovery might be negatively regulated by CD4^+^ CD8^+^ double-positive T cells (58). Similar results were obtained by Dudakov et al. (59), suggesting that cTECs and mTEC^lo^ subsets were the major contributors to TEC recovery. Dumont-Lagacé et al. (60) used the tetracycline-inducible H2B-GFP mouse model to tag slow-cycling label-retaining cells (LRCs), which hypothetically label quiescent stem cells in the adult thymus. Following induction of acute injury by exposure of mice to irradiation, UEA^−^ LRCs displayed a significant increase in proliferation (60). Interestingly, these LRCs were localized in the cortical-medullary junction and were proposed as adult progenitors (60). Recently, Lepletier et al. (61) used the aging/sex steroid-ablation model to demonstrate that during recovery, there was a decrease in the ratio of MHCII^lo^ cTEC to MHCII^hi^ cTEC, followed by a decrease in the ratio of mTEC^lo^ to mTEC^hi^. Taken together, these studies consistently showed that cTECs sharing similar phenotypes to the putative adult bipotent progenitors of TECs can initiate thymic recovery from stress, and potentially could contain a subpopulation of adult progenitors (25, 26).

### Molecular Mechanisms of TEC Regeneration

Many soluble factors are involved in endogenous regeneration of TECs after damage. Following sublethal irradiation, depletion of radiosensitive CD4^+^ CD8^+^ double-positive T cells triggers radioresistant innate lymphoid cells to produce IL-22 (59, 62, 63). IL-22 binding to the IL-22 receptor on TECs then promotes TEC recovery through phosphorylation of STAT3 and STAT5 (59, 63). Of note, cTECs and mTEC^lo^, but not mTEC^hi^ subsets showed significant early responses to administration of IL-22 during recovery (59).

An alternative mechanism of recovery involves keratinocyte growth factors (KGF). KGF is mainly expressed by fibroblasts and its cognate receptor, FgfR2IIIb, is exclusively expressed by TECs in the thymus (64–67). Importantly, administration of KGF to mice exposed to irradiation accelerated the recovery of TECs by enhancing their proliferation (67).

Bone morphogenetic protein 4 (BMP4) also participates in mediating thymic regeneration. BMP4 is predominantly expressed by fibroblasts and radioresistant endothelial cells, and its expression increases soon after radiation exposure (68). Remarkably, administration of thymus-derived *ex vivo*-propagated endothelial cells, but not of endothelial cells derived from other organs, rescued the damage to TECs in mice exposed to radiation (68). Such rescue was driven by increased proliferation of cTECs, reflecting their higher expression of the non-redundant receptor, BMPR2, compared with mTECs (68).

## Conclusions and Future Perspectives

**Figure 1:**
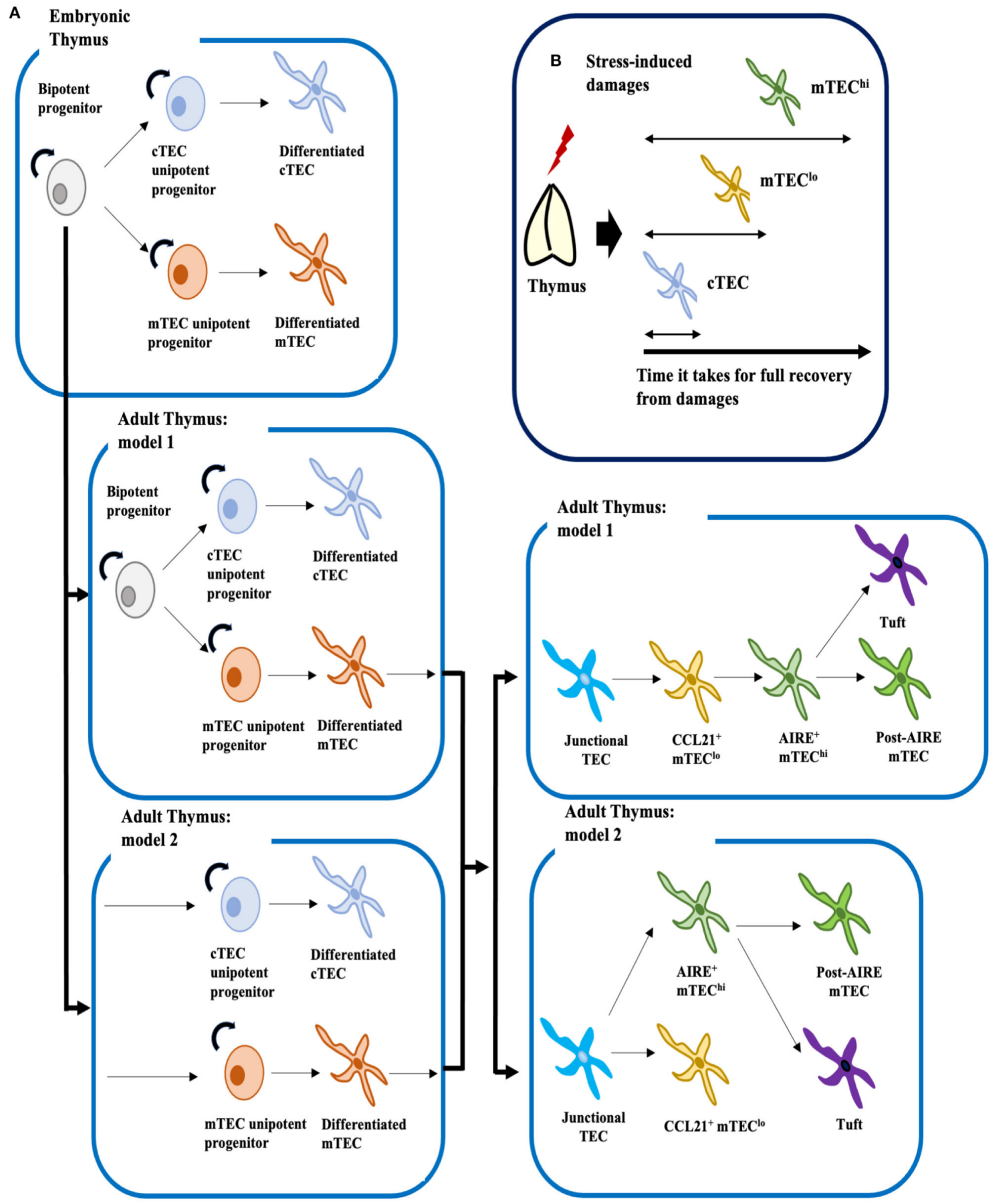
Proposed mechanistic models for development of TECs under homeostatic conditions, and cellular mechanisms of TEC recovery after insults. **(A)** Embryonic bipotent progenitors in the thymus can generate both mature cTECs and mTECs. In the adult thymus, two models for development of cTECs and mTECs can be proposed. The first model, by analogy to the fetal thymus, posits that LY51^+^ MHCII^lo^ or LY51^+^ PLET1^+^ MHCII^hi^ adult bipotent progenitors, though their identities remain controversial, supply both mature cTECs and mTECs. The second model suggests that adult bipotent progenitors are lost with age, and that unipotent progenitors committed to either the cTEC or mTEC lineages supply mature cTECs and mTECs. However, these two models are not necessary mutually exclusive. Similarly, two models can be proposed for development of mTEC^lo^ and mTEC^hi^: one supports linear differentiation from junctional TECs to mature mTEC^hi^ via CCL21^+^ mTEC^lo^, and the other suggests bifurcation of junctional TECs into mature CCL21^+^ mTEC^lo^ and AIRE^+^ mTEC^hi^. Mature AIRE^+^ mTEC^hi^ would then differentiate into Post-AIRE mTECs or tuft cells (4, 35) **(B)** Mouse models of thymic stress have shown that cTECs are the first cell type to recover from insults, followed by mTEC^lo^ and then mTEC^hi^. Therefore, it is possible that the adult progenitor population may be confined to cTECs.

Despite striking progress owing to advances in scRNA-seq technology, the exact mechanisms by which TECs develop remain far from clear, especially in postnatal and adult thymus. Based on our current understanding, we can propose two potential models for the development of cTECs and mTECs (Figure 1A). Given that early progenitors in the adult could arise from β5t^+^ embryonic bipotent progenitors, mature cTECs and mTECs could be replenished by bipotent progenitors or/and by two types of unipotent progenitors separately committed to the cTEC or mTEC lineages (8, 9, 11, 23). Studies of TEC development under homeostatic and recovery conditions suggest that these early progenitors bear cTEC phenotypes (Figure 1B) (25, 26, 58–61).

Recent findings also suggest that similar to mTEC^hi^, mTEC^lo^ could also contain functionally mature cells (30, 31, 34). Thus, two models can be proposed for development of the mTEC lineage: one supports bifurcation from junctional TECs into terminally differentiated mTEC^hi^ and CCL21^+^ mTEC^lo^, and the other, which is grounded in the traditional view that mTEC^lo^ are immature, supports the linear differentiation pathway from junctional TECs to mature mTEC^hi^ via CCL21^+^ mTEC^lo^ (Figure 1A). Further experimentation will be necessary to verify developmental trajectories proposed from *in silico* analyses. Notably, cell type clusters identified in scRNA-seq analyses by Baran-Gale et al. (38) contained mixtures of cells from TEC subsets defined by flow cytometry. Hence, new cell markers will be needed to equate cytometrically defined cell types with the computationally derived cell clusters.

Another key point is the possibility that progenitors identified under homeostatic conditions could differ from those present during TEC recovery. Interestingly, whereas IL-22 is critical for TEC recovery from radiation-inducing damage, deletion of IL-22 did not significantly affect TEC cellularity in steady state conditions. This implies that the TEC progenitor potential may depend on thymic microenvironments. Moreover, the discrepancy in the two studies reporting bipotent adult TEC progenitors may be explained by possible variations and plasticity of TEC progenitors. Indeed, in other epithelial tissues such as the small intestine, skin, mammary gland, and lung, the potential of stem cells appears to change depending on the microenvironment (e.g., niches) and conditions in tissues (69). Cells that lack stemness during steady-state conditions have the capacity to acquire features of stem cells under different settings (70). To resolve similar complexity of TEC progenitors, an *in vivo* fate-mapping study using a specific progenitor marker driving CRE should be useful.

Finally, identification of TEC progenitors has crucial clinical implications. For example, therapeutic targeting of these cells would provide new opportunities for reversal of thymic aging, thereby boosting the responsiveness of an individual's immune system. Ultimately, such a discovery could also contribute to the field of regenerative medicine by allowing creation of thymic organoids, which could open up a new avenue of treatment for immunological disorders.

## Author Contributions

TI wrote the first draft of the manuscript and NA and TA critically reviewed the manuscript. All authors contributed to the article and approved the submitted version.

## Conflict of Interest

The authors declare that the research was conducted in the absence of any commercial or financial relationships that could be construed as a potential conflict of interest.

## References

[1] Miller J. The golden anniversary of the thymus. Nat Rev Immunol. (2011) 11:489–95. 10.1038/nri2993 21617694

[2] Abramson J Anderson G. Thymic epithelial cells. Annu Rev Immunol. (2017) 35:85–118. 10.1146/annurev-immunol-051116-052320 28226225

[3] Wang HX Pan W Zheng L Zhong XP Tan L Liang Z. Thymic epithelial cells contribute to thymopoiesis and T cell development. Front Immunol. (2019) 10:3099. 10.3389/fimmu.2019.03099 32082299 PMC7005006

[4] Kadouri N Nevo S Goldfarb Y Abramson J. Thymic epithelial cell heterogeneity: TEC by TEC. Nat Rev Immunol. (2020) 20:239–53. 10.1038/s41577-019-0238-0 31804611

[5] Anderson MS Venanzi ES Klein L Chen ZB Berzins SP Turley SJ. Projection of an immunological self shadow within the thymus by the aire protein. Science. (2002) 298:1395–401. 10.1126/science.1075958 12376594

[6] Rossi SW Jenkinson WE Anderson G Jenkinson EJ. Clonal analysis reveals a common progenitor for thymic cortical and medullary epithelium. Nature. (2006) 441:988–91. 10.1038/nature04813 16791197

[7] Bleul CC Corbeaux T Reuter A Fisch P Mönting JS Boehm T. Formation of a functional thymus initiated by a postnatal epithelial progenitor cell. Nature. (2006) 441:992–6. 10.1038/nature04850 16791198

[8] Baik S Jenkinson EJ Lane PJL Anderson G Jenkinson WE. Generation of both cortical and Aire(+) medullary thymic epithelial compartments from CD205(+) progenitors. Eur J Immunol. (2013) 43:589–94. 10.1002/eji.201243209 23299414 PMC3960635

[9] Gill J Malin M Hollander GA Boyd R. Generation of a complete thymic microenvironment by MTS24(+) thymic epithelial cells. Nat Immunol. (2002) 3:635–42. 10.1038/ni812 12068292

[10] Bennett AR Farley A Blair NF Gordon J Sharp L Blackburn CC. Identification and characterization of thymic epithelial progenitor cells. Immunity. (2002) 16:803–14. 10.1016/S1074-7613(02)00321-7 12121662

[11] Ohigashi I Zuklys S Sakata M Mayer CE Zhanybekova S Murata S. Aire-expressing thymic medullary epithelial cells originate from beta 5t-expressing progenitor cells. Proc Natl Acad Sci U S A. (2013) 110:9885–90. 10.1073/pnas.1301799110 23720310 PMC3683726

[12] Ribeiro AR Rodrigues PM Meireles C Di Santo JP Alves NL. Thymocyte selection regulates the homeostasis of IL-7-expressing thymic cortical epithelial cells *in vivo*. J Immunol. (2013) 191:1200–9. 10.4049/jimmunol.1203042 23794633

[13] Meireles C Ribeiro AR Pinto RD Leitão C Rodrigues PM Alves NL. Thymic crosstalk restrains the pool of cortical thymic epithelial cells with progenitor properties. Eur J Immunol. (2017) 47:958–69. 10.1002/eji.201746922 28318017

[14] Gray DH Seach N Ueno T Milton MK Liston A Lew AM. Developmental kinetics, turnover, and stimulatory capacity of thymic epithelial cells. Blood. (2006) 108:3777–85. 10.1182/blood-2006-02-004531 16896157

[15] Hamazaki Y Fujita H Kobayashi T Choi Y Scott HS Matsumoto M. Medullary thymic epithelial cells expressing Aire represent a unique lineage derived from cells expressing claudin. Nat Immunol. (2007) 8:304–11. 10.1038/ni1438 17277780

[16] Sekai M Hamazaki Y Minato N. Medullary thymic epithelial stem cells maintain a functional thymus to ensure lifelong central T cell tolerance. Immunity. (2014) 41:753–61. 10.1016/j.immuni.2014.10.011 25464854

[17] Gray D Abramson J Benoist C Mathis D. Proliferative arrest and rapid turnover of thymic epithelial cells expressing Aire. J Exp Med. (2007) 204:2521–8. 10.1084/jem.20070795 17908938 PMC2118482

[18] Rode I Boehm T. Regenerative capacity of adult cortical thymic epithelial cells. Proc Natl Acad Sci U S A. (2012) 109:3463–8. 10.1073/pnas.1118823109 22331880 PMC3295321

[19] Yano M Kuroda N Han H Meguro-Horike M Nishikawa Y Kiyonari H. Aire controls the differentiation program of thymic epithelial cells in the medulla for the establishment of self-tolerance. J Exp Med. (2008) 205:2827–38. 10.1084/jem.20080046 19015306 PMC2585853

[20] White AJ Nakamura K Jenkinson WE Saini M Sinclair C Seddon B. Lymphotoxin signals from positively selected thymocytes regulate the terminal differentiation of medullary thymic epithelial cells. J Immunol. (2010) 185:4769–76. 10.4049/jimmunol.1002151 20861360 PMC3826119

[21] Wang X Laan M Bichele R Kisand K Scott HS Peterson P. Post-Aire maturation of thymic medullary epithelial cells involves selective expression of keratinocyte-specific autoantigens. Front Immunol. (2012) 3:19. 10.3389/fimmu.2012.00019 22448160 PMC3310317

[22] Metzger TC Khan IS Gardner JM Mouchess ML Johannes KP Krawisz AK. Lineage tracing and cell ablation identify a post-Aire-expressing thymic epithelial cell population. Cell Rep. (2013) 5:166–79. 10.1016/j.celrep.2013.08.038 24095736 PMC3820422

[23] Ohigashi I Zuklys S Sakata M Mayer CE Hamazaki Y Minato N. Adult thymic medullary epithelium is maintained and regenerated by lineage-restricted cells rather than bipotent progenitors. Cell Rep. (2015) 13:1432–43. 10.1016/j.celrep.2015.10.012 26549457

[24] Mayer CE Žuklys S Zhanybekova S Ohigashi I Teh HY Sansom SN Shikama-Dorn N. Dynamic spatio-temporal contribution of single β5t+ cortical epithelial precursors to the thymus medulla. Eur J Immunol. (2016) 46:846–56. 10.1002/eji.201545995 26694097 PMC4832341

[25] Ulyanchenko S O'Neill KE Medley T Farley AM Vaidya HJ Cook AM. Identification of a bipotent epithelial progenitor population in the adult thymus. Cell Rep. (2016) 14:2819–32. 10.1016/j.celrep.2016.02.080 26997270 PMC4819909

[26] Wong K Lister NL Barsanti M Lim JMC Hammett MV Khong DM. Multilineage potential and self-renewal define an epithelial progenitor cell population in the adult thymus. Cell Rep. (2014) 8:1198–209. 10.1016/j.celrep.2014.07.029 25131206

[27] Derbinski J Gäbler J Brors B Tierling S Jonnakuty S Hergenhahn M. Promiscuous gene expression in thymic epithelial cells is regulated at multiple levels. J Exp Med. (2005) 202:33–45. 10.1084/jem.20050471 15983066 PMC2212909

[28] Gäbler J Arnold J Kyewski B. Promiscuous gene expression and the developmental dynamics of medullary thymic epithelial cells. Eur J Immunol. (2007) 37:3363–72. 10.1002/eji.200737131 18000951

[29] Rossi SW Kim MY Leibbrandt A Parnell SM Jenkinson WE Glanville SH. RANK signals from CD4(+)3(–) inducer cells regulate development of Aire-expressing epithelial cells in the thymic medulla. J Exp Med. (2007) 204:1267–72. 10.1084/jem.20062497 17502664 PMC2118623

[30] Lucas B White AJ Cosway EJ Parnell SM James KD Jones ND. Diversity in medullary thymic epithelial cells controls the activity and availability of iNKT cells. Nat Commun. (2020) 11:2198. 10.1038/s41467-020-16041-x 32366944 PMC7198500

[31] Kozai M Kubo Y Katakai T Kondo H Kiyonari H Schaeuble K. Essential role of CCL21 in establishment of central self-tolerance in T cells. J Exp Med. (2017) 214:1925–35. 10.1084/jem.20161864 28611158 PMC5502431

[32] Onder L Nindl V Scandella E Chai Q Cheng HW Caviezel-Firner S. Alternative NF-κB signaling regulates mTEC differentiation from podoplanin-expressing precursors in the cortico-medullary junction. Eur J Immunol. (2015) 45:2218–31. 10.1002/eji.201545677 25973789

[33] Bornstein C Nevo S Giladi A Kadouri N Pouzolles M Gerbe F. Single-cell mapping of the thymic stroma identifies IL-25-producing tuft epithelial cells. Nature. (2018) 559:622–6. 10.1038/s41586-018-0346-1 30022162

[34] Dhalla F Baran-Gale J Maio S Chappell L Holländer GA Ponting CP. Biologically indeterminate yet ordered promiscuous gene expression in single medullary thymic epithelial cells. Embo J. (2020) 39:e101828. 10.15252/embj.2019101828 31657037 PMC6939203

[35] Miller CN Proekt I von Moltke J Wells KL Rajpurkar AR Wang H. Thymic tuft cells promote an IL-4-enriched medulla and shape thymocyte development. Nature. (2018) 559:627–31. 10.1038/s41586-018-0345-2 30022164 PMC6062473

[36] Miragaia RJ Zhang X Gomes T Svensson V Ilicic T Henriksson J. Single-cell RNA-sequencing resolves self-antigen expression during mTEC development. Sci Rep. (2018) 8:685. 10.1038/s41598-017-19100-4 29330484 PMC5766627

[37] La Manno G Soldatov R Zeisel A Braun E Hochgerner H Petukhov V. RNA velocity of single cells. Nature. (2018) 560:494–8. 10.1038/s41586-018-0414-6 30089906 PMC6130801

[38] Baran-Gale J Morgan MD Maio S Dhalla F Calvo-Asensio I Deadman ME. Ageing compromises mouse thymus function and remodels epithelial cell differentiation. Elife. (2020) 9:e56221. 10.7554/eLife.56221 32840480 PMC7490013

[39] Wells KL Miller CN Gschwind AR Wei W Phipps JD Anderson MS. Combined transient ablation and single-cell RNA-sequencing reveals the development of medullary thymic epithelial cells. Elife. (2020) 9:e60188. 10.7554/eLife.60188 33226342 PMC7771965

[40] Park JE Botting RA Domínguez Conde C Popescu DM Lavaert M Kunz DJ. A cell atlas of human thymic development defines T cell repertoire formation. Science. (2020) 367:eaay3224. 10.1101/2020.01.28.911115 32079746 PMC7611066

[41] Chaudhry MS Velardi E Dudakov JA van den Brink RM. Thymus: the next (re)generation. Immunol Rev. (2016) 271:56–71. 10.1111/imr.12418 27088907 PMC4837659

[42] Kinsella S Dudakov JA. When the damage is done: injury and repair in thymus function. Front Immunol. (2020) 11:1745. 10.3389/fimmu.2020.01745 32903477 PMC7435010

[43] Horie K Kato T Kudo T Sasanuma H Miyauchi M Akiyama N. Impact of spaceflight on the murine thymus and mitigation by exposure to artificial gravity during spaceflight. Sci Rep. (2019) 9:19866. 10.1038/s41598-019-56432-9 31882694 PMC6934594

[44] Fiume G Scialdone A Albano F Rossi A Tuccillo FM Rea D. Impairment of T cell development and acute inflammatory response in HIV-1 Tat transgenic mice. Sci Rep. (2015) 5:13864. 10.1038/srep13864 26343909 PMC4561375

[45] Hick RW Gruver AL Ventevogel MS Haynes BF Sempowski GD. Leptin selectively augments thymopoiesis in leptin deficiency and lipopolysaccharide-induced thymic atrophy. J Immunol. (2006) 177:169–76. 10.4049/jimmunol.177.1.169 16785512 PMC1993881

[46] Wang SD Huang KJ Lin YS Lei HY. Sepsis-induced apoptosis of the thymocytes in mice. J Immunol. (1994) 152:5014–21. 8176219

[47] Wyllie H. Glucocorticoid-induced thymocyte apoptosis is associated with endogenous endonuclease activation. Nature. (1980) 284:555–6. 10.1038/284555a0 6245367

[48] Yuuki H Yoshikai Y Kishihara K Matsuzaki G Ayukawa K Nomoto K. The expression and sequences of T cell antigen receptor beta-chain genes in the thymus at an early stage after sublethal irradiation. J Immunol. (1989) 142:3683–91. 2523931

[49] Zúñiga-Pflücker JC Jiang D Schwartzberg PL Lenardo MJ. Sublethal gamma-radiation induces differentiation of CD4–/CD8– into CD4+/CD8+ thymocytes without T cell receptor beta rearrangement in recombinase activation gene 2–/– mice. J Exp Med. (1994) 180:1517–21. 10.1084/jem.180.4.1517 7931082 PMC2191689

[50] Ivanov VN Nikolić-Zugić J. Biochemical and kinetic characterization of the glucocorticoid-induced apoptosis of immature CD4+CD8+ thymocytes. Int Immunol. (1998) 10:1807–7. 10.1093/intimm/10.12.1807 9885901

[51] Goldberg GL King CG Nejat RA Suh DY Smith OM Bretz JC. Luteinizing hormone-releasing hormone enhances T cell recovery following allogeneic bone marrow transplantation. J Immunol. (2009) 182:5846–54. 10.4049/jimmunol.0801458 19380833 PMC2760441

[52] Heng TS Goldberg GL Gray DH Sutherland JS Chidgey AP Boyd RL. Effects of castration on thymocyte development in two different models of thymic involution. J Immunol. (2005) 175:2982–93. 10.4049/jimmunol.175.5.2982 16116185

[53] Sutherland JS Goldberg GL Hammett MV Uldrich AP Berzins SP Heng TS. Activation of thymic regeneration in mice and humans following androgen blockade. J Immunol. (2005) 175:2741–53. 10.4049/jimmunol.175.4.2741 16081852

[54] Lai KP Lai JJ Chang P Altuwaijri S Hsu JW Chuang KH. Targeting thymic epithelia AR enhances T-cell reconstitution and bone marrow transplant grafting efficacy. Mol Endocrinol. (2013) 27:25–37. 10.1210/me.2012-1244 23250486 PMC3545211

[55] Zoller AL Kersh GJ. Estrogen induces thymic atrophy by eliminating early thymic progenitors and inhibiting proliferation of beta-selected thymocytes. J Immunol. (2006) 176:7371–8. 10.4049/jimmunol.176.12.7371 16751381

[56] Jaffe HL. The influence of the suprarenal gland on the thymus: I. regeneration of the thymus following double suprarenalectomy in the rat. J Exp Med. (1924) 40:325–42. 10.1084/jem.40.3.325 19868921 PMC2128579

[57] Miller JF. Immunological function of the thymus. Lancet. (1961) 2:748–9. 10.1016/S0140-6736(61)90693-6 14474038

[58] Kaneko KB Tateishi R Miyao T Takakura Y Akiyama N Yokota R. Quantitative analysis reveals reciprocal regulations underlying recovery dynamics of thymocytes and thymic environment in mice. Commun Biol. (2019) 2:444. 10.1038/s42003-019-0688-8 31815199 PMC6884561

[59] Dudakov JA Hanash AM Jenq RR Young LF Ghosh A Singer NV. Interleukin-22 drives endogenous thymic regeneration in mice. Science. (2012) 336:91–5. 10.1126/science.1218004 22383805 PMC3616391

[60] Dumont-Lagacé M Gerbe H Daouda T Laverdure JP Brochu S Lemieux S. Detection of quiescent radioresistant epithelial progenitors in the adult thymus. Front Immunol. (2017) 8:1717. 10.3389/fimmu.2017.01717 29259606 PMC5723310

[61] Lepletier A Hun ML Hammett MV Wong K Naeem H Hedger M. Interplay between Follistatin, Activin A, and BMP4 signaling regulates postnatal thymic epithelial progenitor cell differentiation during aging. Cell Rep. (2019) 27:3887–901.e4. 10.1016/j.celrep.2019.05.045 31242421

[62] Pan B Liu J Zhang Y Sun Y Wu Q Zhao K. Acute ablation of DP thymocytes induces up-regulation of IL-22 and Foxn1 in TECs. Clin Immunol. (2014) 150:101–8. 10.1016/j.clim.2013.11.002 24333537

[63] Pan B Wang D Li L Shang L Xia F Zhang F. IL-22 accelerates thymus regeneration via Stat3/Mcl-1 and decreases chronic graft-versus-host disease in mice after allotransplants. Biol Blood Marrow Transplant. (2019) 25:1911–19. 10.1016/j.bbmt.2019.06.002 31195136

[64] Rossi S Blazar BR Farrell CL Danilenko DM Lacey DL Weinberg KI. Keratinocyte growth factor preserves normal thymopoiesis and thymic microenvironment during experimental graft-versus-host disease. Blood. (2002) 100:682–91. 10.1182/blood.V100.2.682 12091365

[65] Min D Taylor PA Panoskaltsis-Mortari A Chung B Danilenko DM Farrell C. Protection from thymic epithelial cell injury by keratinocyte growth factor: a new approach to improve thymic and peripheral T-cell reconstitution after bone marrow transplantation. Blood. (2002) 99:4592–600. 10.1182/blood.V99.12.4592 12036893

[66] Alpdogan O Hubbard VM Smith OM Patel N Lu S Goldberg GL. Keratinocyte growth factor (KGF) is required for postnatal thymic regeneration. Blood. (2006) 107:2453–60. 10.1182/blood-2005-07-2831 16304055 PMC1895735

[67] Rossi SW Jeker LT Ueno T Kuse S Keller MP Zuklys S. Keratinocyte growth factor (KGF) enhances postnatal T-cell development via enhancements in proliferation and function of thymic epithelial cells. Blood. (2007) 109:3803–11. 10.1182/blood-2006-10-049767 17213286 PMC1874572

[68] Wertheimer T Velardi E Tsai J Cooper K Xiao S Kloss CC. Production of BMP4 by endothelial cells is crucial for endogenous thymic regeneration. Sci Immunol. (2018) 3:eaal2736. 10.1126/sciimmunol.aal2736 29330161 PMC5795617

[69] Blanpain C Fuchs E. Stem cell plasticity. Plasticity of epithelial stem cells in tissue regeneration. Science. (2014) 344:1242281. 10.1126/science.1242281 24926024 PMC4523269

[70] Snippert HJ Clevers H. Tracking adult stem cells. EMBO Rep. (2011) 12:113–22. 10.1038/embor.2010.216 21252944 PMC3049439

